# Both interferon alpha and lambda can reduce all intrahepatic HDV infection markers in HBV/HDV infected humanized mice

**DOI:** 10.1038/s41598-017-03946-9

**Published:** 2017-06-16

**Authors:** Katja Giersch, Maria Homs, Tassilo Volz, Martina Helbig, Lena Allweiss, Ansgar W. Lohse, Jörg Petersen, Maria Buti, Teresa Pollicino, Camille Sureau, Maura Dandri, Marc Lütgehetmann

**Affiliations:** 1 I. Department of Internal Medicine, University Medical Hospital Hamburg-Eppendorf, Hamburg, Germany; 2 Hospital Vall d’Hebron, Barcelona, Spain; 3 German Center for Infection Research (DZIF), Hamburg-Lübeck-Borstel site, Hamburg, Germany; 4 IFI Institute for Interdisciplinary Medicine at Asklepios Clinic St. Georg, Hamburg, Germany; 5 Department of Human Pathology, University of Messina, Messina, Italy; 6 Institut National de la Transfusion Sanguine, Paris, France; 7 Institute of Microbiology, Virology and Hygiene, University Medical Hospital Hamburg-Eppendorf, Hamburg, Germany

## Abstract

Co-infection with hepatitis B (HBV) and D virus (HDV) is associated with the most severe course of liver disease. Interferon represents the only treatment currently approved. However, knowledge about the impact of interferons on HDV in human hepatocytes is scant. Aim was to assess the effect of pegylated interferon alpha (peg-IFNα) and lambda (peg-IFNλ), compared to the HBV-polymerase inhibitor entecavir (ETV) on all HDV infection markers using human liver chimeric mice and novel HDV strand-specific qRT-PCR and RNA *in situ* hybridization assays, which enable intrahepatic detection of HDV RNA species. Peg-IFNα and peg-IFNλ reduced HDV viremia (1.4 log and 1.2 log, respectively) and serum HBsAg levels (0.9-log and 0.4-log, respectively). Intrahepatic quantification of genomic and antigenomic HDV RNAs revealed a median ratio of 22:1 in untreated mice, resembling levels determined in HBV/HDV infected patients. Both IFNs greatly reduced intrahepatic levels of genomic and antigenomic HDV RNA, increasing the amounts of HDAg- and antigenomic RNA-negative hepatocytes. ETV-mediated suppression of HBV replication (2.1-log) did not significantly affect HBsAg levels, HDV productivity and/or release. In humanized mice lacking adaptive immunity, IFNs but not ETV suppressed HDV. Viremia decrease reflected the intrahepatic reduction of all HDV markers, including the antigenomic template, suggesting that intracellular HDV clearance is achievable.

## Introduction

Around 15 million people worldwide are chronically infected with hepatitis Delta virus (HDV). Persistent co-infection with hepatitis B virus (HBV) and HDV leads to the most severe form of viral hepatitis, which is associated with an accelerated course of liver fibrosis and cirrhosis^1^. Nucleosid(t)e analogs (NUCs, e.g. lamivudine, adefovir, entecavir), which potently inhibit the HBV reverse transcriptase and are approved antiviral drugs for the treatment of chronic HBV infection, do not show beneficial effects in chronically HBV/HDV co-infected patients^2^. To date, no HDV-specific therapy is available or has been approved^3^. Thus, treatment options for chronically HBV/HDV co-infected patients are limited to interferons and pegylated interferon alpha (peg-IFNα) is the only currently approved treatment. Unfortunately, therapy outcomes with peg-IFNα (after 48 weeks of treatment) are unsatisfactory, as evidenced by the low rates of sustained virological responses, HDV relapses after treatment cessation^4^ and the occurrence of complicating side effects. A few clinical studies suggest that prolonging the IFN treatment to several years might lead to higher response rates^5^. Although peg-IFNα is able to reduce HDV viremia during treatment, knowledge about its antiviral effects or those of other potential therapeutic cytokines on HDV in infected cells is still scant.

HDV is a circular, negative, single-stranded RNA virus with a genome of 1,679 nucleotides in length. HDV replication occurs in the nucleus of hepatocytes in a double rolling circle process^6^ and leads to the accumulation of three different HDV RNAs: the genomic RNA, antigenomic RNA and mRNA^7^. The antigenomic HDV RNA is the central template for the transcription of full length genomic RNA and the hepatitis delta antigen (HDAg) mRNA. Newly produced virions consist of genomic HDV RNA, which is associated with HDAg proteins and surrounded by surface antigens of the hepatitis B virus (HBsAg). The HDAg proteins exist in two different isoforms: a 24 kDa small isoform, which is needed for replication, and the 27 kDa isoform generated as a consequence of an adenosine deaminase acting on RNA (ADAR)-mediated RNA editing event, which inhibits HDV RNA replication and drives viral assembly^8, 9^. The balance between genomic and antigenomic RNA appears to be crucial to allow persistence of HDV infection and is highly regulated by the two forms of HDAg, as well as through epigenetic modifications^10, 11^.

Due to the limited availability of experimental HBV/HDV infection models, knowledge about the behavior and stability of genomic and antigenomic RNAs in HBV/HDV co-infection and upon therapy is limited, although it may be determined to improve current treatment regiments.

The aim of this study was to investigate the impact of both pegylated interferon alpha and lambda, which were reported to have similar antiviral effects in chronic HBV infected patients^12^, on HDV replication *in vivo*, by using human liver chimeric mice^13^ and focussing on intrahepatic changes of the different HDV infection markers. The capacity of the two therapeutic cytokines to reduce HDV was compared to treatment with the nucleotide analogs entecavir (ETV). In particular, we investigated the effects of the different treatments in HBV/HDV co-infected human hepatocytes by establishing a new magnetic beads-based qRT-PCR assay enabling specific quantification of both genomic and antigenomic HDV RNA forms in chimeric mice and patient liver samples, as well as a RNA *in situ* hybridization technique allowing the analysis of HDV RNA expression at single cell level.

## Results

### Peg-IFNs but not ETV treatment decreased HDV viremia

**Figure 1:**
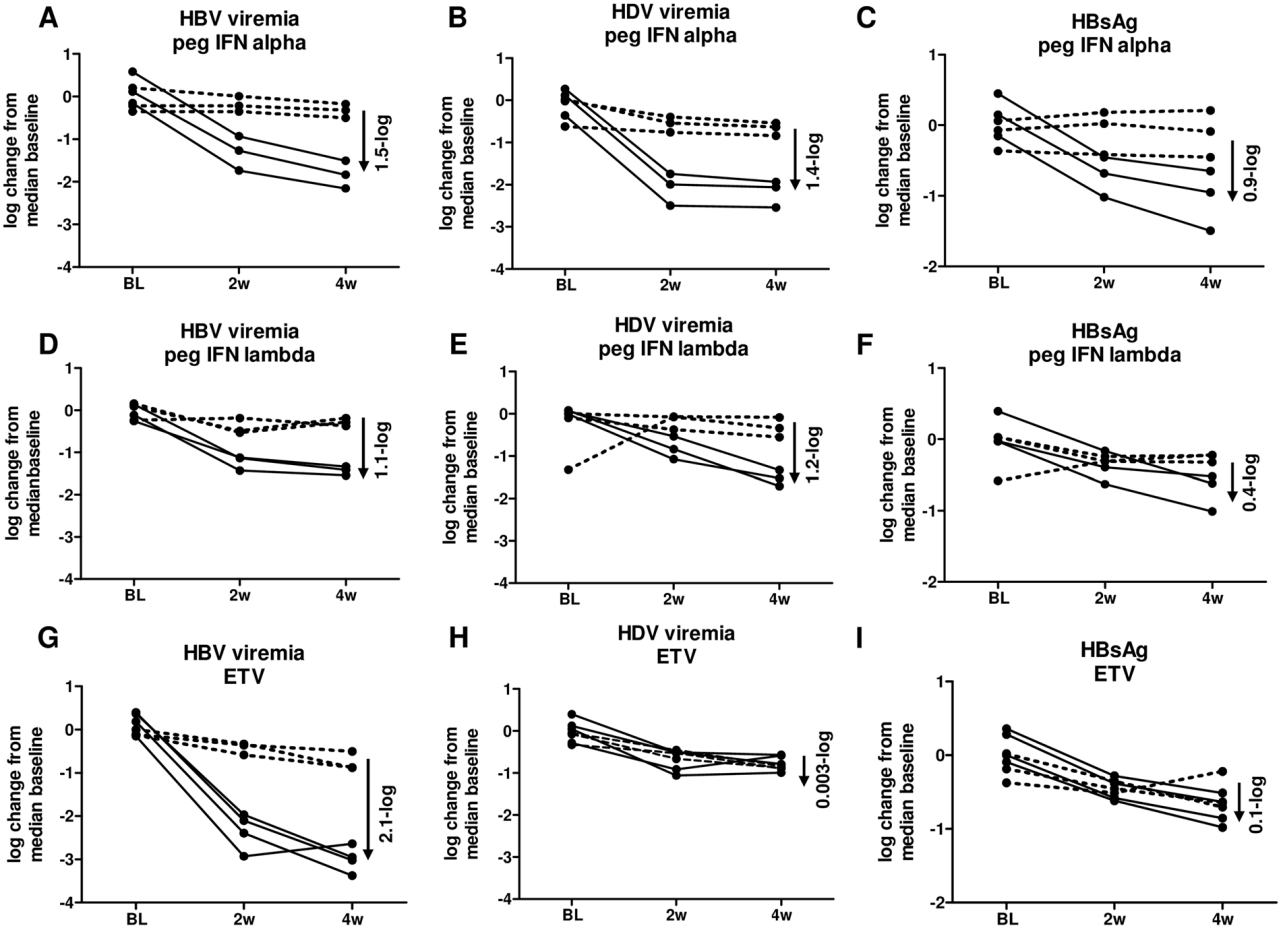
Viremia changes upon treatment. Depicted is HBV viremia (**A,D,G**), HDV viremia (**B,E,H**) and levels of circulating HBsAg (**C,F,I**) as fold change from median baseline levels from each mouse, which was either treated (solid line) with peg-INFα (25ng/g body weight) (**A–C**), peg-INFλ (25ng/g body weight) (**D–F**) or ETV (1 µg/ml supplemented in drinking water) (**G–I**) or left untreated (dashed line). Baseline levels were obtained at time-points where HBV/HDV co-infection was stable; the median baseline level was calculated from all mice (treated and untreated) for each study. The fold reduction is shown on each graph as log-change and was calculated as median of the control versus median of the treated group at the end of treatment.

Stably HBV/HDV co-infected human chimeric UPA/SCID/beige (USB) mice displaying comparable viremia levels received peg-IFNα (25ng/g body weight, n = 3), peg-IFNλ (25ng/g body weight, n = 3) or ETV (1 µg/ml supplemented in drinking water, n = 4) for four weeks or remained untreated as controls (n = 3 for each treatment group). Figure 1 shows the HBV viremia, HDV viremia and circulating HBsAg levels in treated and untreated mice as log change from median baseline levels of each mouse. At the end of antiviral treatment (week four), peg-IFNα induced a median 1.5-log and 1.4-log decrease in HBV and HDV viremia, respectively (Fig. 1A,B), while HBsAg levels were decreased by median 0.9-log compared to median levels obtained from untreated controls at the same time-point (Fig. 1C). Humanized mice treated with peg-IFNλ showed a similar reduction of HBV (median 1.1-log, Fig. 1D) and HDV viremia (median 1.2-log, Fig. 1E), but a less pronounced reduction of circulating HBsAg levels (median 0.4-log Fig. 1F). Daily administration of ETV led to a stronger decrease of HBV viremia (median 2.1-log) (Fig. 1G), whereas HDV viremia (Fig. 1H) and HBsAg levels (Fig. 1I) did not change substantially (median 0.003-log and median 0.1-log reduction, respectively) after four weeks of treatment compared to median levels determined in matched control animals.

### Peg-IFNs provoked a strong reduction of intrahepatic HDAg

**Figure 2:**
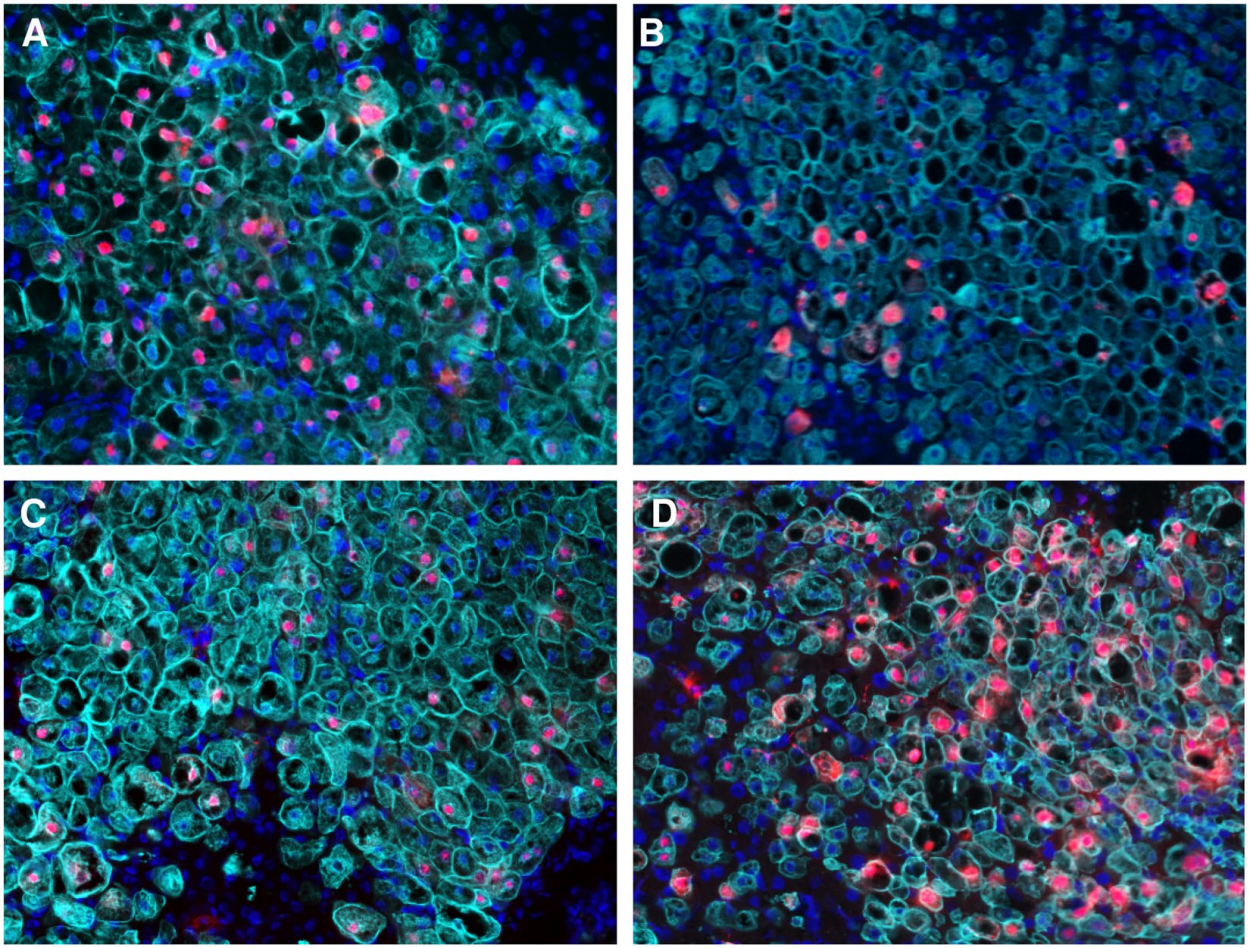
Intrahepatic HDAg changes upon treatment. Immunofluorescence staining of HDAg (red) and human hepatocytes (CK18, light blue) in humanized liver specimen of untreated control mice (**A**) or mice either treated with peg-INFα (**B**), peg-INFλ (**C**) or ETV (**D**). Nuclei are stained in dark blue (Hoechst 33258).

In line with virological parameters, immunofluorescence staining of liver specimens from mice sacrificed at the end of the experiment revealed that the amount of HDAg-positive human hepatocytes was clearly reduced by the interferon-based treatments but not in ETV-treated mice. 60.2% (range = 51.0–76.4%) of human hepatocytes appeared HDAg-positive in control animals (Fig. 2A), while only 9.6% (range = 4.7–18.3%) stained HDAg-positive in peg-IFNα treated mice (Fig. 2B) and 14.7% (range = 10.1–25.2%) in peg-IFNλ treated mice (Fig. 2C). In contrast, the amount of HDAg-positive human hepatocytes remained similar to controls in ETV-treated mice (51.2%, range = 41.3–63.6%, Fig. 2D).

### Establishment of a new biotinylated magnetic beads (BMB) qRT-PCR based assay for genomic and antigenomic HDV RNA quantification

**Figure 3:**
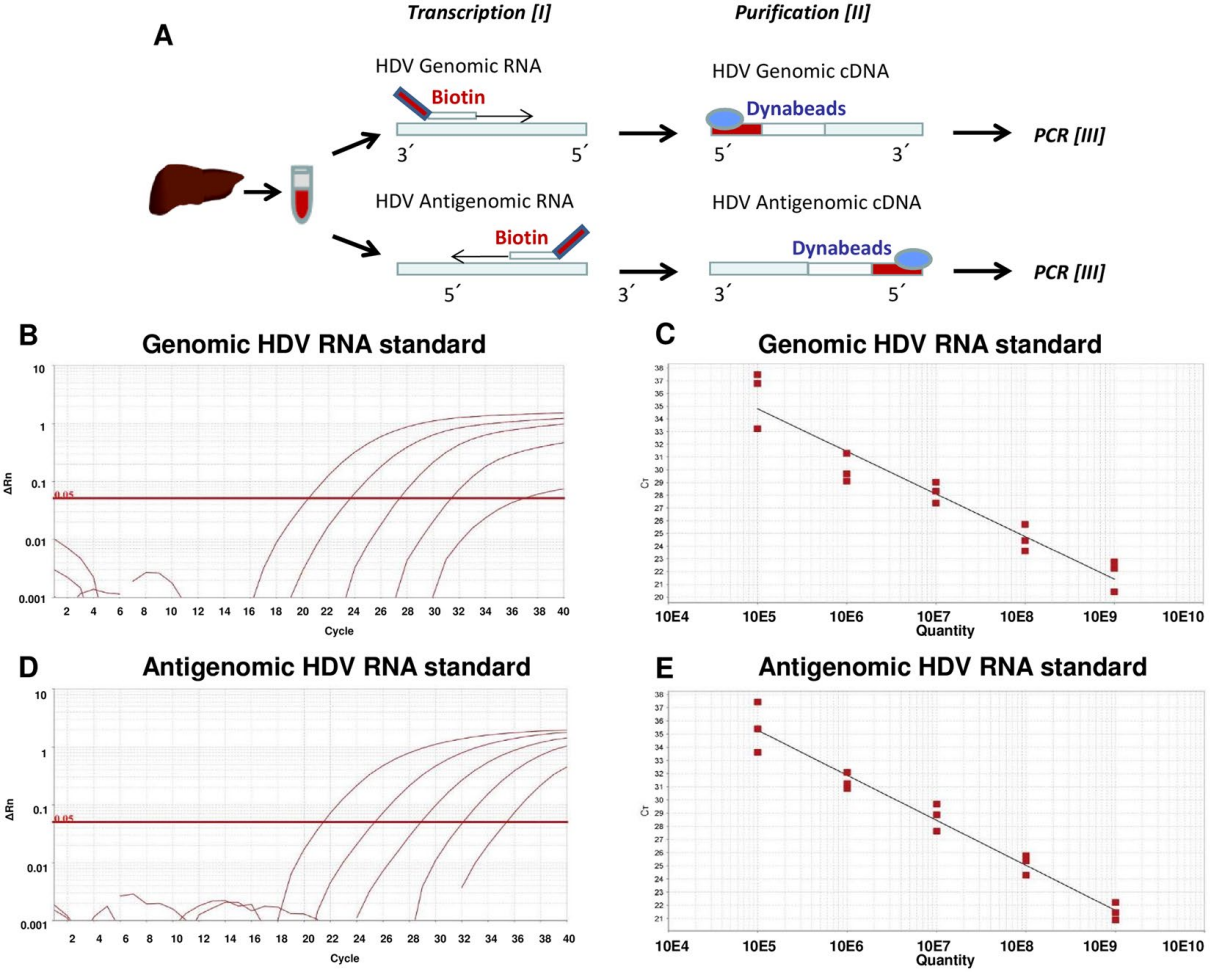
Establishment of strand-specific biotinylated magnetic beads (BMB)-based qRT-PCR assay and genomic/antigenomic HDV RNA standard curves. (**A**) HDV RNAs were separately reverse transcribed using biotinylated HDV specific genomic or antigenomic primer, purified with magnetic beads and then quantified by performing a qRT-PCR. B-E) Standard curves of genomic HDV RNA with a slope of −3.351 and an efficiency of 98.8% (**B**) and antigenomic HDV RNA with a slope of −3.417 and an efficiency of 96.2% (**D**). Amplification curves of 10^9^, 10^8^, 10^7^, 10^6^ and 10^5^ copies genomic (**C**) and antigenomic (**E**) HDV RNA.

As depicted in Fig. 3A, intrahepatic amounts of genomic and antigenomic HDV RNA were determined using a novel strand specific qRT-PCR assay. Briefly, total RNA extracted from liver tissues or serum was first incubated with a biotinylated primer specific for either genomic or antigenomic HDV RNA^14^. After reverse transcription, biotinylated cDNA was purified via magnetic beads exclusively binding the biotinylated cDNA products which were then quantified by qRT-PCR^14^. For the strand-specific quantification of genomic and antigenomic HDV RNAs, artificial full-length genomic and antigenomic HDV RNA standards were generated as described in Supplementary Material and Methods. The standard curves of genomic HDV RNA showed a slope of −3.351 and 98.8% efficiency (Fig. 3B,C), and a slope of −3.417 and an efficiency of 96.2% for the antigenomic HDV RNA (Fig. 3D,E). The analyses determining the specificity of this assay are described in Supplementary Results. Of note, ratios of genomic to antigenomic HDV RNA can be quantified up to 1:100 without crosstalk. At ratios above 1:100 the assay becomes unspecific by detecting genomic HDV RNA when using the antigenomic HDV RNA primer (suppl. results and suppl. Fig. S1).

**Figure 4:**
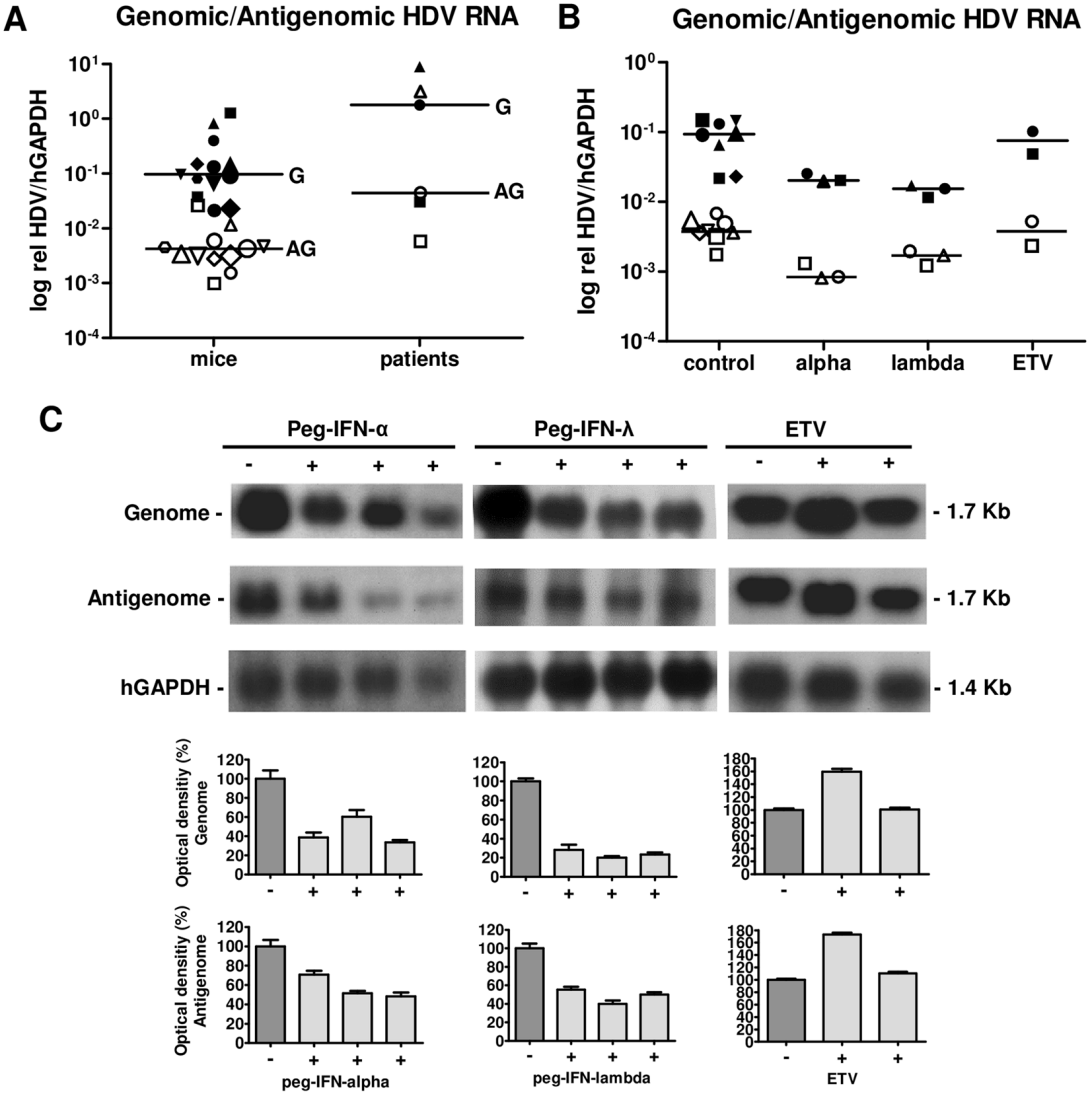
Genomic and antigenomic HDV RNA. Amounts of genomic (black symbols) and antigenomic (white symbols) HDV RNA detected with the novel BMB qRT-PCR assay are similar in patients liver biopsies (n = 3) and humanized USB mice (n = 13) (**A**). Peg-INFα (n = 3) and peg-INFλ (n = 3) treatment reduced amounts of genomic (black symbols) and antigenomic (white symbols) HDV RNA relative to hGAPDH clearly, while ETV (n = 2) did not show a reduction of HDV RNA levels compared to untreated control mice (n = 8) (**B**). Same symbols within one group (e.g. circle, triangle) represent distinct values of the same mouse/patient (**A, B**). (**C**) Shows representative northern blot analyses and their densitometric quantification of signal bands for genomic (1.7 kb) and antigenomic HDV RNA (1.7 kb) from untreated, peg-IFNα, peg-INFλ and ETV treated mice.

In stably HBV/HDV co-infected, humanized USB mice (n = 13, median HDV viremia 8.5 × 10^6^ copies/ml) a median of 0.097 genomic HDV RNA and 0.004 antigenomic HDV RNA relative to human glyceraldehyde 3-phosphate dehydrogenase (hGAPDH) were detected (Fig. 4A). The ratio of genomic to antigenomic HDV RNA ranged from 8 to 73:1 (median: 22:1). Interestingly, ratios of genomic to antigenomic HDV RNA in a similar range (3–41:1) were obtained in a first proof-of-concept analysis of human liver biopsies from three patients with chronic HBV/HDV infections (Fig. 4A).

### Peg-IFNs but not ETV decreased both intrahepatic HDV RNA and HBV RNA levels

**Figure 5:**
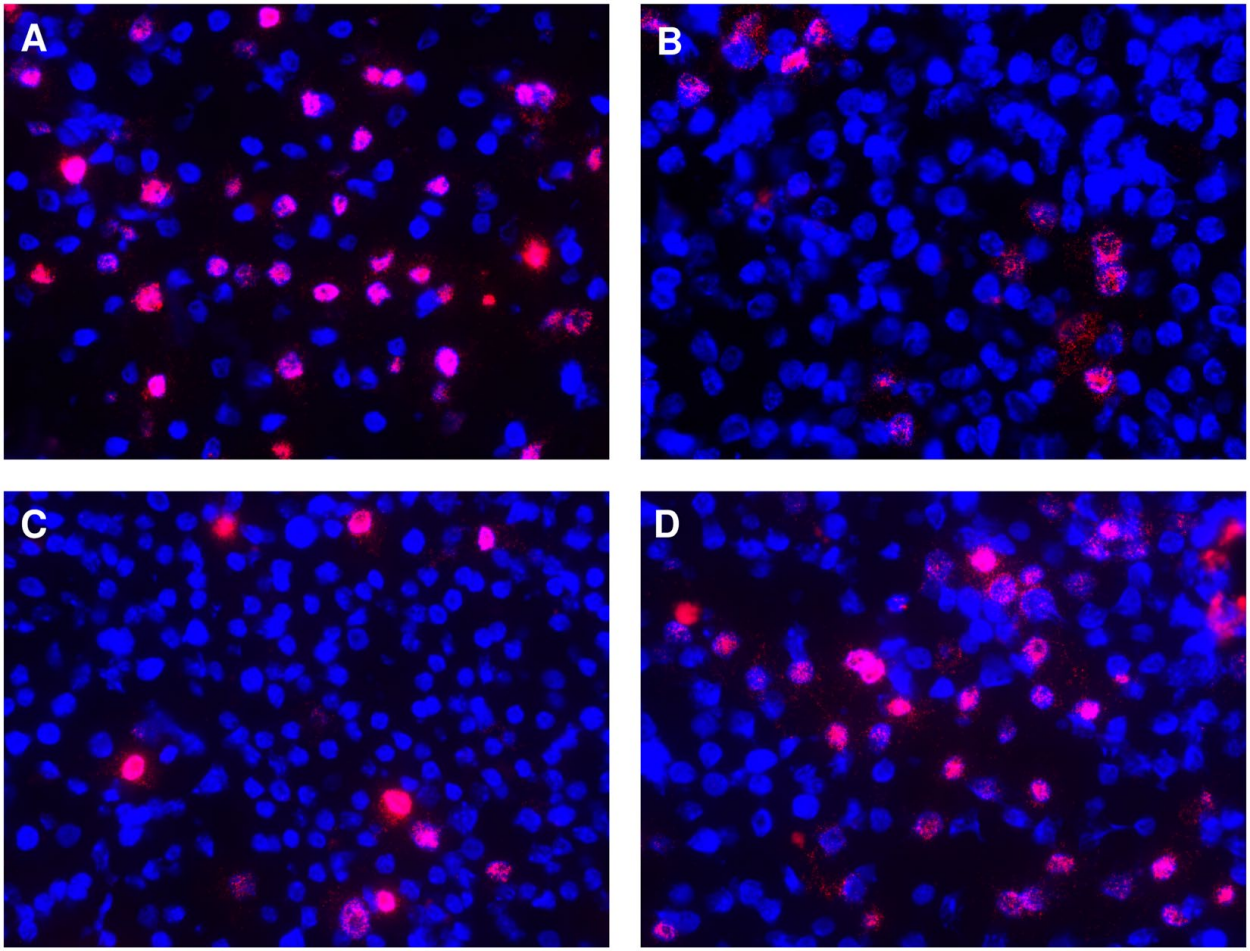
Antigenomic HDV RNA determined by an RNA *in situ* hybridisation assay (RNAScope). Immunofluorescence staining of both antigenomic HDV RNA and HDV mRNA (red dots) in humanized liver specimen of untreated control mice (**A**) and mice either treated with peg-INFα (**B**), peg-INFλ (**C**) or ETV (**D**). Nuclei are stained in dark blue (Hoechst 33258).

To evaluate changes of intrahepatic HDV replication induced by the different antiviral compounds, treated and untreated human chimeric USB mice were sacrificed at the end of treatment and genomic and antigenomic HDV RNA levels were analyzed. Figure 4B shows that peg-IFNα and peg-INFλ reduced both genomic and antigenomic HDV RNA relative to hGAPDH in comparison to untreated controls. Both genomic and antigenomic HDV RNAs were decreased 4.6-/4.4-fold and 6.1/2.2-fold upon peg-IFNα and peg-IFNλ treatment, respectively, but only 1.4/1.1-fold in ETV treated mice. Notably, the ratios of genomic to antigenomic HDV RNA remained constant in all groups (median control: 18:1; peg-IFNα: 28:1; peg-IFNλ: 11:1; ETV: 24:1). The results obtained by using the BMB qRT-PCR assay were confirmed by northern blot analyses (Fig. 4C). Densitometric quantification of northern blot bands revealed that - similarly to qRT-PCR analyses - administration of peg-IFNα and peg-IFNλ but not of ETV clearly lowered amounts of both genomic and antigenomic HDV RNA. Moreover, we performed an RNA *in situ* hybridisation assay (RNAScope) to visualize both antigenomic HDV RNA and HDV mRNA replicative intermediates in liver specimens at a single cell level (Fig. 5). Interestingly, single cell analysis revealed that the amount of antigenomic HDV RNA and HDV mRNA positive cells appeared clearly decreased in livers of peg-IFNα (Fig. 5B **)** and peg-IFNλ (Fig. 5C **)** treated mice compared to uninfected (Fig. 5A **)** or ETV treated animals (Fig. 5D **)**, suggesting that both IFNs are able to clear HDV in a proportion of infected hepatocytes.

Total HBV RNA levels relative to hGAPDH appeared reduced 15.0-fold with peg-IFNα and 8.6-fold upon treatment with peg-IFNλ in the setting of HBV/HDV co-infection (suppl. Fig. S2). In line with our previous study^15^, total HBV RNA levels remained unchanged in ETV-treated mice.

### Interferon-stimulated genes in untreated and treated humanized mice

**Figure 6:**
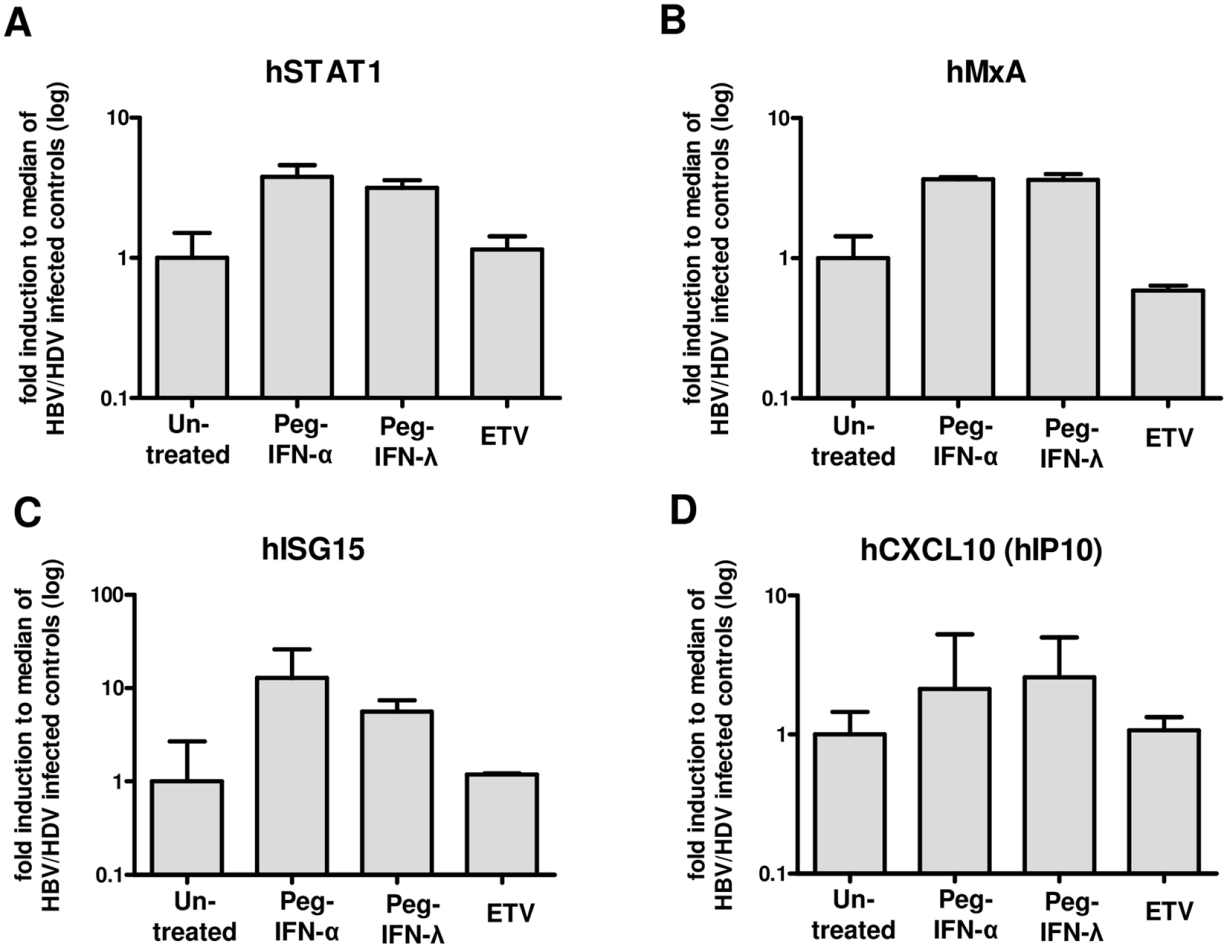
Interferon-stimulated genes. Expression of human specific signalling genes (hSTAT 1, (**A**), interferon-stimulated genes (hMxA, (**B**) hISG15, (**C**) and cytokines (hCXCL10, (**D**) in HBV/HDV infected untreated and peg-IFNα, peg-IFNλ and ETV treated animals. Expression levels are relative the median of two housekeeping genes (hGAPDH, hRPL30). Depicted is the log fold induction (median and range) from median of untreated controls.

To analyze whether treatment with different interferons enabled the induction of innate immune responses in a comparable manner, human genes involved in innate immune signalling were analyzed by qRT-PCR in livers of HBV/HDV co-infected mice treated with peg-IFNα, peg-IFNλ and ETV. Compared to untreated control animals both peg-IFNα and peg-IFNλ showed a clear and similar induction of human signal transducer and activator of transcription (hSTAT1) (peg-IFNα: 3.8x; peg-IFN: 3.2x), human myxovirus resistance A (hMxA) (peg-IFNα: 3.7x; peg-IFNλ: 3.6x), human interferon stimulated gene 15 (hISG15) (peg-IFNα: 12.9x; peg-IFNλ: 5.6x) and human C-X-C motif chemokine 10 (hCXCL10) (peg-IFNα: 2.2x; peg-IFNλ: 2.6x) (Fig. 6). Interestingly, the induction of human genes involved in innate immunity appeared comparable to that determined in uninfected mice, which received a single injection of peg-IFNα or peg-IFNλ, indicating that HDV does not restrict the IFN signalling induced by pegylated IFNs (suppl. Fig. S3). As previously reported^15^, ETV did not interfere with innate immunity as seen by unchanged levels of hSTAT1 (fold induction: 1.1x), hMxA (0.6x), hISG15 (1.2x) and hCXCL10 (1.1x) when compared to untreated mice (Fig. 6).

## Discussion

The knowledge of the intrahepatic virological changes induced by different antiviral treatments in HBV/HDV co-infected patients is still limited. Both the availability of robust experimental models able to recapitulate the entire life cycle of HBV and HDV in infected hepatocytes and the establishment of sensitive molecular assays are necessary to gain insights about the impact of distinct drugs on HDV replication. In this study, we comparatively analyzed the antiviral effects of peg-IFNα, peg-IFNλ and ETV *in vivo* by employing HBV/HDV co-infected human liver chimeric mice. Moreover, we established and validated innovative magnetic beads-based qRT-PCR and RNA *in situ* hybridization assays to specifically quantify and visualize the different HDV RNA forms at single cell level in infected livers. We show that treatment of mice lacking adaptive immune responses with either peg-IFNα or peg-IFNλ for four weeks significantly lowered HDV viremia and amounts of HDAg-positive human hepatocytes. Previous reports have shown that IFNα and - to some extent - also IFNλ can mediate HBV suppression in infected cells by acting through different mechanisms^15–18^. However, to our knowledge this is the first study demonstrating that both peg-IFNα and peg-IFNλ can exert potent serological and intrahepatic anti-HDV effects *in vivo*, without the involvement of adaptive immune responses. It should also be noted that due to impaired lymphoid development, the activity of important innate components, like natural killer (NK) cells, are also strongly reduced in these humanized mice. Clinical observations indicated that peg-IFNλ administration provoked fewer side-effects than peg-IFNα^19^ and might therefore represent an attractive therapeutic alternative to peg-IFNα in chronic HDV infection, especially if prolonged regimens extending treatment duration beyond 48 weeks shall be employed in the attempt to increase response rates^5^. While peg-IFNλ is already used in clinical development for chronically HDV infected patients (phase II clinical trials by Eiger BioPharmaceuticals, Palo Alto, CA; ClinicalTrials.gov identification number: NCT02765802), this study underlines the importance to investigate the intrahepatic activity of different therapeutics that are emerging from preclinical development. Four-week administration of ETV strongly reduced HBV viremia and intrahepatic HBV DNA levels, but had no impact on circulating HBsAg, HDV viremia and intrahepatic HDV loads. These results are in line with data from clinical trials showing that 1-year treatment with peg-IFN-α^4^ but not with nucleoside analogs, like ETV^20^, reduced HDV viremia in infected patients^20^.

Active HDV replication occurs in the nucleus of hepatocytes using a double rolling circle process^6^ and leads to the accumulation of three different HDV RNAs: the genomic RNA, antigenomic RNA and mRNA^7^. To dissect the antiviral effects of these three different compounds on intrahepatic HDV replication, we established a novel magnetic beads-based qRT-PCR based assay allowing the specific determination of genomic and antigenomic HDV RNAs. In the past, the differentiation between the genomic and antigenomic HDV RNA forms was attainable only by northern blot^21^. However, northern blot analyses are time-consuming, have lower sensitivity and are only semi-quantitative. In 2008, Tseng *et al*. proposed to quantify genomic and antigenomic HDV RNA by performing reverse transcription with nucleotide tagged HDV strand-specific primers followed by RT-PCR^22^. However, such qRT-PCR procedures may also generate false-positive PCR products since genomic and antigenomic HDV RNA are exact complements of each other and therefore may lead to self-priming and self-annealing. Our novel BMB qRT-PCR assay includes an additional purification step based on the use of magnetic beads, which is essential to circumvent self-priming and self-annealing of genomic and antigenomic HDV RNA. Moreover, the sequence of the HDV-specific primers and probes used in the BMB assay have been shown to detect all HDV genotypes^14^, thus making the assay useful for clinical trials or cohort studies.

To our knowledge, quantitative estimations of genomic and antigenomic HDV RNA in the liver of patients infected with HDV have not been available and could only be estimated in woodchucks and chimpanzees^21^. Proof of concept analyses performed in liver biopsies derived from chronically HBV/HDV infected patients using this BMB qRT-PCR assay indicated that genomic HDV RNAs were 3 to 41-fold more abundant than the amounts of antigenomic HDV RNA. Notably, these ranges are comparable to ratios obtained in livers of humanized USB mice (8 to 73 more abundance of genomic to antigenomic HDV RNA) and are also in line with previous studies detecting ratios of genomic to antigenomic HDV RNA in woodchucks and chimpanzees (5–22:1) by northern blot analysis^21^. Furthermore, this new BMB qRT-PCR assay is able to specifically detect genomic to antigenomic HDV RNAs up to a ratio of 1:100. This range appears sufficient to cover the ratios existing in hepatocytes of humans, woodchucks and chimpanzees.

In this study, we observed that both IFNs, but not ETV, led to a clear and comparable decrease of both genomic and antigenomic HDV RNA levels, while ratios of genomic to antigenomic HDV RNA were maintained in all treated and control groups. The strong decrease of both HDV RNA forms could also be confirmed by northern blot and by applying a new highly sensitive RNA *in situ* hybridization assay (RNAScope), which permitted specific visualization of the HDV replicative antigenomic intermediates at single cell level. It is worth noting that in interferon-treated animals a large proportion of the human hepatocytes appeared both HDAg- and HDV RNA- negative, thus suggesting that HDV infection could be purged at least in a proportion of human hepatocytes after four weeks of peg-IFN treatment.

Interestingly, short-term treatment with IFNα had no antiviral effect on HDV replication *in vitro* ^23^ and another study even indicated that HDV can hinder IFN-induced nuclear STAT1 translocation^24^. Accordingly, we previously showed that HBV can limit nuclear STAT1 translocation and enhancement of human ISGs upon treatment with regular IFNα *in vivo* ^25^. However, prolonged therapy with higher doses of peg-IFNα was able to break such restriction in HBV-infected humanized mice^15^. Therefore, it is plausible that four-week treatment of HBV/HDV co-infected mice with peg-IFNs could also overcome the observed blockades of IFN signaling, since both peg-IFNs led to a similar and clear induction of human genes, which are involved in innate immune signaling (e.g. classic interferon stimulated genes (ISGs) and chemokines), providing evidence that ISG induction and antiviral activity could be elicited even in the absence of an adaptive immune system^15^. Previous cell culture analyses already suggested that signaling through either the IFNα or IFNλ receptor complex results in the activation of the same janus kinase (JAK)-STAT signal transduction cascade^26^, and accordingly, the RNA expression of human STAT1 was similarly induced in human hepatocytes of mice that were either treated with peg-IFNα or peg-IFNλ, but not with ETV. Consequently, the biological activities induced by either type I or type III IFNs seem to be similar^26^. However, the molecular mechanisms of IFN-induced HDV suppression *in vivo* are still unclear and needs to be addressed in further studies.

In conclusion, this study demonstrates that the antigenomic HDV RNA, which acts as template of viral replication, could be significantly reduced by peg-IFN treatment. Although the rates of sustained virological responses (SVR) in chronic HDV infected patients treated with peg-IFNα are low and viral relapse is observed in a high percentage of patients^4^, our study suggests that peg-IFN-based therapies may still play a supportive role in combination with new therapeutic approaches involving, for instance, HDV entry^13, 27^, assembly^28^ or HBsAg release^29^ inhibitors. Finally, this study underlines the importance of investigating the intrahepatic effects provoked by new treatment approaches and provides new molecular tools enabling the quantification of genomic and antigenomic HDV RNA contents in future preclinical and clinical trials.

## Material and Methods

### Generation of humanized USB mice and viral infection

Human liver chimeric USB (urokinase-type plasminogen activator (uPA)/severe combined immunodeficiency (SCID)/beige) mice were generated by transplanting one million thawed cryo-preserved human hepatocytes obtained from two different human donors (both with intermediate interferon responsive C/T IL28 locus) into homozygous USB mice, as previously reported^13^. Primary human hepatocytes were isolated from rejected explant livers using protocols approved by the Ethical Committee of the city and state of Hamburg (OB-042/06) and accorded to the principles of the Declaration of Helsinki. Mice were maintained under specific pathogen free conditions in accordance with institutional guidelines under approved protocols. Repopulation rates were estimated by human serum albumin (HSA) concentrations in mouse sera (Bethyl Laboratories, Biomol GmbH, Hamburg, Germany) and confirmed at sacrifice by determining human cell contents by histology and qRT-PCR using the beta-globin gene kit (Roche DNA control Kit; Roche Diagnostics)^13^. Animals displaying high levels of human chimerism (>2 mg/ml HSA in serum) were used for the study. To establish an HBV/HDV co-infection, human chimeric USB mice received a single peritoneal injection of 10 µl HBV- and HDV-positive, patient derived serum (5 × 10^6^ HBV DNA copies/mouse, genotype D, HBeAg-positive; 5 × 10^6^ HDV RNA copies/mouse, genotype 1). The inoculum corresponded to a MOI of approximately 0.2 for both viruses, by estimating an average of 3 × 10^7^ human hepatocytes per mouse liver^30^. Blood samples were taken every other week as indicated in the results. Mice were sacrificed at the end of treatment. Liver specimens removed at sacrifice were cryo-conserved in chilled isopentane for further histological and molecular analyses. All animal experiments were conducted in accordance with the European Communities Council Directive (86/609/EEC) and were approved by the City of Hamburg, Germany.

### Antiviral Treatments

Stably HBV/HDV co-infected (>10 weeks post virus inoculation) human-chimeric USB mice were either treated with peg-IFNα (Pegasys, provided by Hoffmann-La Roche Inc., Basel, Switzerland) (n = 3), peg-IFNλ (provided by Bristol-Myers Squibb, CT US) (n = 3) or with ETV (n = 4) for four weeks. Uninfected mice were treated once with peg-IFNα or peg-IFNλ. All animals (four-week dose or single dose) were sacrificed 24 hours after their last interferon injection for intrahepatic analyses. The IFNs were injected subcutaneously twice a week (each 25 ng/g body weight)^15^. In humans, 180 µg of either peg-IFNα or peg-IFNλ are used for a single injection, which would be an equivalent of 50ng/20 g mouse. According to commonly used dose scaling to adjust human doses to mouse equivalent doses and in line with previous reports^15, 17^ we used 500ng/20 g mouse (25ng/g body weight) in this study. For ETV treatment, mice received drinking water supplemented with 1 μg/ml Baraclude Solution (Bristol-Myers Squibb, Munich, Germany)^15^. Control mice were infected in parallel, but left untreated (n = 3 for each treatment arm).

### Human liver samples

Cryo-preserved human liver biopsies were obtained from three chronically HBV/HDV-infected patients. The biological samples were obtained for diagnostic purpose. The surplus material and patients characteristics were used in accordance to the principles of the Declaration of Helsinki, after informed consent of patients and protocols were approved by the ethical committee of the city and state of Hamburg (PV4081).

### Virological measurements and intrahepatic quantification in cryo-preserved livers

Viral DNA and RNA were extracted from serum samples using the QiAmp MinElute Virus Spin kit (Qiagen, Hilden, Germany) and from liver tissues using the RNeasy RNA purification kit (Qiagen, Hilden, Germany). HDV viremia and intrahepatic HDV RNA levels were determined by reverse transcription and qRT-PCR using the ABI Fast 1-Step Virus Master (Applied Biosystems, Carlsbad, USA), HDV specific primers and probes^14^ on a ABI Viia7 (Applied Biosystems, Carlsbad, USA). In detail, RNA extracted from 5 μl mouse serum or 1 μl liver derived RNA were denatured at 95 °C for 10 min, immediately cooled down to −20 °C and reverse transcribed at 50 °C for 5 min. After inactivation of the reverse transcriptase at 95 °C for 20 s, amplification was performed under the following conditions: initial step 95 °C 20 s, 40 cycles at 95 °C for 3 s and 60 °C for 30 s. HBV viremia was determined as reported^30^. Known amounts of HBV or HDV containing plasmids were used as standards for quantification and hGAPDH for expression normalization^21^. HBsAg quantification was performed using the Architect HBsAg assay (Abbott Ireland Diagnostics, Sligo, Ireland).

### Biotinylated magnetic beads-based qRT-PCR assay (BMB assay)

RNA extracted from 10 μl mouse serum or 1 μg liver derived RNA was denatured at 95 °C for 10 min with 0.5 μM of a biotinylated HDV specific forward primer (biotin-GCGCCGGCYGGGCAAC) for genomic HDV RNA quantification or a biotinylated HDV specific reverse primer (biotin-TTCCTCTTCGGGTCGGCATG) for antigenomic HDV RNA quantification^14^ and immediately cooled down to −20 °C. After addition of a ABI Fast 1-Step Virus Master (Applied Biosystems, Carlsbad, USA) to a final volume of 20 µl, reverse transcription was performed at 50 °C for 5 min on an ABI Viia7 (Applied Biosystems, Carlsbad, USA) and enzymes were inactivated at 95 °C for 20 s. Biotinylated cDNA was purified with the MinElute PCR Purification Kit (Qiagen, Hilden, Germany) and isolated with dynabeads specifically interacting with biotin (Dynal Kilobase Binder Kit, Invitrogen, Darmstadt, Germany) following the manufacturer’s instructions. In brief, 5 μl dynabeads were washed and re-suspended in Binding Buffer, incubated with 20 μl biotinylated cDNA for 3 hours, then washed twice with Washing Buffer and once with sterile, RNase free water. For qRT-PCR 1 μl of purified biotinylated cDNA bound to dynabeads, HDV specific primers and probes^14^ and an ABI Fast Advanced Master (Applied Biosystems, Carlsbad, USA) were used under the following conditions: Initial step 95 °C 20 s; 40 cycles at 95 °C for 3 s and 60 °C for 30 s. hGAPDH (Applied Biosystems, Hs99999905_m1) was used for normalization of intrahepatic HDV RNAs. The BMB assay was established and validated using a genomic and antigenomic HDV RNA standard (generation of these standards is described in Supplementary Material and Methods).

### Northern blot

Total RNA was extracted from liver tissue specimens with the TRIzol reagent (Life Technologies, Carlsbad, USA) by following the manufacturer’s instructions. Extracted RNA was further purified using the RNeasy spin columns (Qiagen, Hilden, Germany). RNA concentration was measured using an ND-1000 spectrophotometer (NanoDrop Technologies, Wilmington, DE, USA) at 260 nm. RNA quality and quantity were monitored by ethidium bromide staining and by UV absorbance. Northern blot analysis was performed following standard procedures as previously described^31^. Radioactive probes were prepared by using a random priming protocol utilizing full-length HDV cDNA or hGAPDH cDNA templates and deoxycytidine triphosphate labeled on the alpha phosphate group with 32 P.

(32 P α-dCTP) (Amersham, New Jersey, USA). In particular, HDV cDNA templates specific for genomic and antigenomic HDV RNAs were obtained by the use of Transcriptor First strand cDNA synthesis (Roche, Basel, Switzerland) and HDV specific primers. Monomers of genomic and antigenomic HDV RNAs were transcribed from pHDVII- a pGEM-T easy vector (Promega, Wisconsin, USA) with dual-opposed promoters containing a 980 bp HDV fragment (from nt 305 to nt 1285)- with T7 and SP6 MEGAscript kit (Ambion, Carlsbad, USA) after linearization by HindII and XbaI digestion, respectively. Densitometric quantitation of signal bands for genomic (1.7 kb) and antigenomic HDV RNA (1.7 kb) from untreated and treated mice was performed by Quantity One 1-D Analysis Software (BioRad, Laboratories, Hercules, USA); data are from 3 independent experiments and values are expressed as mean ± SD.

### Immunohistochemistry

Cryostat sections of chimeric mouse livers were stained as previously described^13^. Briefly, sections were fixed with acetone and incubated with mouse anti-CK18 (1:400; Dako, Glostrup, Denmark) and human anti-Delta (anti-HDAg-positive human serum, 1:8,000). Specific signals were visualized with Alexa 488- or 546-labeled secondary antibodies (Invitrogen, Darmstadt, Germany). Nuclear staining was achieved by Hoechst 33258 (1:2,000 diluted, Invitrogen, Darmstadt, Germany). Stained sections were then mounted with fluorescent mounting media (Dako, Glostrup, Denmark) and analyzed with the fluorescence microscope BZ9000 (Keyence, Osaka, Japan) using the same settings for the different experimental groups. The percentages of HDAg-positive human hepatocytes were estimated as previously described^13^ and by using 2 visual fields (displaying approximately 500 human hepatocytes each) from 3 different liver sections per mouse.

### RNA *in situ* hybridization (RNAScope)

RNA *in situ* hybridization was performed on paraformaldehyde-fixed, cryo-preserved liver sections using the RNAScope Fluorescent Multiplex Kit (Advanced Cell Diagnostics, ACD, Hayward, CA, USA) according to the manufacturer’s instructions^32^. Briefly, liver sections were dehydrated and pretreated with Pretreat 4 (Pretreatment Kit, ACD) for 30 min. Samples were incubated with specific target probes which specifically bind both antigenomic HDV RNA and HDV mRNA (GT-1, RNAScope assay number 433471) for 2 h at 40 °C (HybEZ oven, ACD). DAPI staining was performed to visualize nuclei. Stained sections were analyzed by fluorescence microscopy (Biorevo BZ-9000, Keyence, Osaka, Japan) using a 60 × /1.40 NA oil objective. Merged z stack images were prepared using the same settings for all groups.

### Expression of human ISGs and cytokines

To determine expression levels of genes related to IFN signaling (ISGs and cytokines) in human hepatocytes repopulating the mouse liver, primers specifically recognizing human transcripts and not cross-reacting with murine genes were used (Taqman Gene Expression Assays, Applied Biosystems). Analyzed were hSTAT1 (assay ID: Hs01013989_m1), hMxA (Hs00895608_m1), hISG15 (Hs00192713_m1) and hIP10/hCXCL10 (Hs00171042_m1). For determining the expression of ISGs and cytokines, RNA was extracted as described above, cDNA was synthesized with the Transcriptor First Strand cDNA Synthesis Kit (Roche, Mannheim, Germany) using oligo-dT primer according to the manufacturer’s instructions and qRT-PCR was performed with the ABI Fast Advanced Master (Applied Biosystems, Carlsbad, USA) in an ABI Viia7 (Applied Biosystems, Carlsbad, USA). Expression levels were calculated relative to the median of hGAPDH and human 60 S ribosomal protein L30 (hRPL30) and are depicted as log change from median values obtained from untreated control mice.

## Electronic supplementary material

Supplementary Material
