# Supplementary material for: Both interferon alpha and lambda can reduce all intrahepatic HDV infection markers in HBV/HDV infected humanized mice
Source: Sci Rep. 2017 Jun 16;7:3757. doi: 10.1038/s41598-017-03946-9 (PMC5473824; doi:10.1038/s41598-017-03946-9)
Supplement: Supplementary file 1 — Supplementary Material [file 41598_2017_3946_MOESM1_ESM.pdf]

# Both interferon alpha and lambda can reduce all markers of HDV infection in HBV/HDV infected humanized mice

Katja Giersch[1], Maria Homs[2], Tassilo Volz[1], Martina Helbig[1], Lena Allweiss[1], Ansgar W. Lohse[1, 3], Jörg Petersen[4], Maria Buti[2], Teresa Pollicino[5], Camille Sureau[6], Maura Dandri[1, 3#*], Marc Lütgehetmann[1,7#*]

1. I. Department of Internal Medicine, University Medical Hospital Hamburg-Eppendorf; Hamburg

2. Hospital Vall d'Hebron, Barcelona, Spain

3. German Center for Infection Research (DZIF), Hamburg-Lübeck-Borstel site, Germany

4. IFI Institute for Interdisciplinary Medicine at Asklepios Clinic St. Georg, Hamburg

5. Department of Human Pathology, University of Messina, Messina, Italy

6. Institut National de la Transfusion Sanguine, Paris, France

7. Institute of Microbiology, Virology and Hygiene, University Medical Hospital Hamburg-Eppendorf, Hamburg

Supplementary Material and Methods

Preparation of the genomic and antigenomic HDV RNA standard. For genomic HDV RNA the HDV full genome plasmid (pBluescript + HDV) was digested with BamHI and in vitro transcribed from the T7 promoter using the MAXIscript kit (Ambion, Darmstadt, Germany). The antigenomic HDV RNA was generated after HindIII digestion of the HDV plasmid and transcription with the T3 promoter. Genomic and antigenomic HDV RNA standards were purified with isopropanol/Trizol (Invitrogen, Darmstadt, Germany) and the Master Pure Complete DNA and RNA purification kit (Epicentre, Madison, USA). For quantification, genomic and antigenomic HDV RNA standards were used and mixed with 1 µg RNA of uninfected livers, imitating real conditions of measurements in livers.

Supplementary Results

Specificity of BMB qRT-PCR assay for genomic and antigenomic HDV RNA quantification. The specificity of the BMB qRT-PCR assay was investigated by the determination of genomic and antigenomic RNA standards (artificial RNA) with the correct primer and the respective opposite primer (genomic primer on antigenomic HDV RNA standard and vice versa) (**suppl. fig. S1A, B**). Unspecific reverse transcription with the opposite primer occurred, though in a very limited extent and without relevance for the natural ratio of genomic and antigenomic RNA in livers. Ratios of genomic to antigenomic RNA can be quantified up to 1:100 without crosstalk (**suppl. fig. S1C**), which was revealed by quantifying artificial mixtures of $5x10^8$ copies genomic HDV RNA with $5x10^8$ (ratio 1:1), $5x10^7$ (ratio 1:10) and $5x10^6$ (ratio 1:100) copies of antigenomic HDV RNA. These mixtures were reverse transcribed with the antigenomic primer and amplification curves showed that the ct value for $5x10^6$ copies antigenomic HDV RNA (in a mixture with $5x10^8$ copies of genomic HDV RNA; 1:100 ratio) is 31, which is in accordance with the unspecific amplification signal for $5x10^8$ copies of pure genomic HDV RNA (also ct value of 31) reverse transcribed with the antigenomic primer, indicating that the antigenomic HDV RNA standard in a mixture with genomic HDV RNA can be detected until a dilution of 1:100 copies (genomic: antigenomic). Moreover, the novel BMB qRT-PCR assay was used to determine specific HDV RNA levels in the serum of a chronically HBV/HDV co-infected humanized USB mouse ($2.4x10^5$ copies/ml) and by detecting genomic but not antigenomic HDV RNA levels in mouse serum (**suppl. fig. S1D**).

Supplementary Figure and Figure Legends

Suppl. figure 1

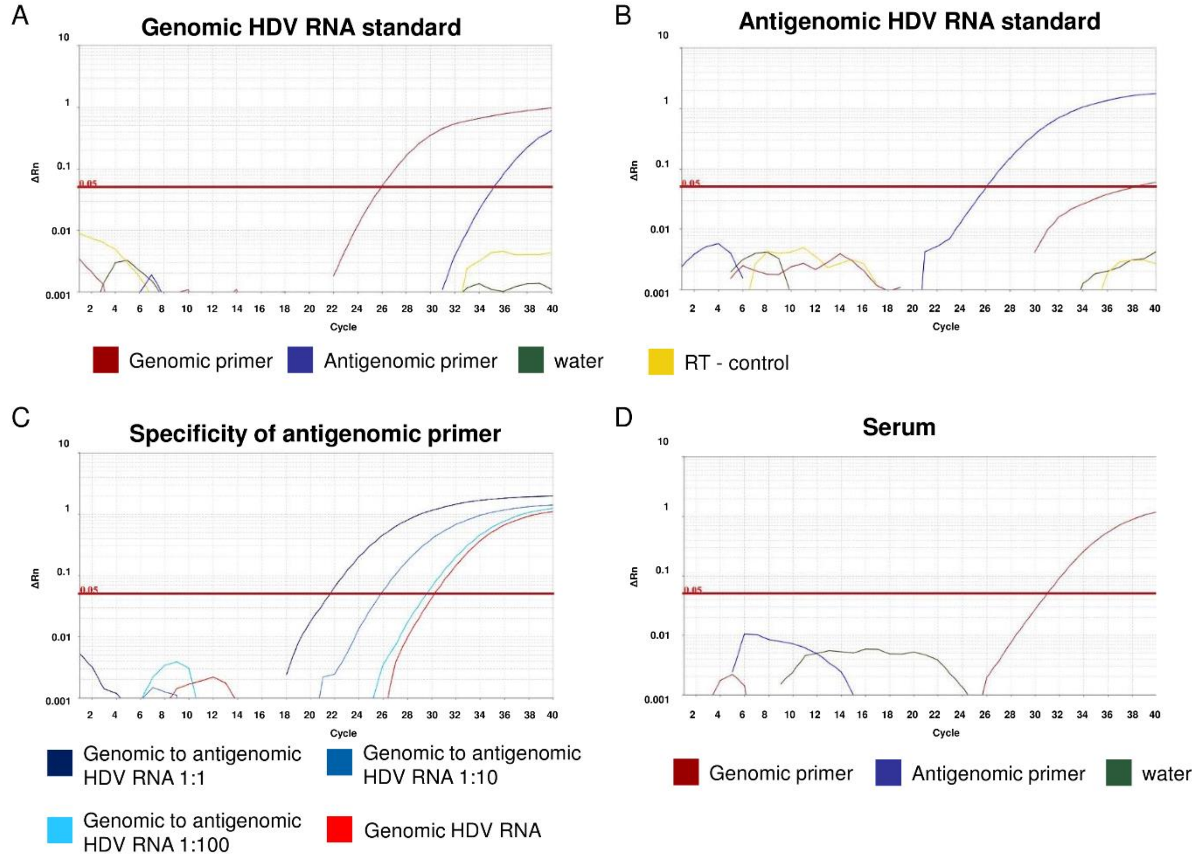

Suppl. figure 1. Specificity of novel BMB qRT-PCR assay. A) Amplicifcation curves of 10^7 copies genomic HDV RNA standard reverse transcribed with the biotinylated HDV genomic (ct=26.0) or antigenomic (ct=34.5) primer. **B)** Amplicifcation curves of 10^7 copies antigenomic HDV RNA standard transcribed with the biotinylated HDV antigenomic (ct=26.0) or genomic (ct=38) primer. **A, B)** As negative controls untranscribed HDV RNA standards (no reverse transcriptase = RT – control) and water were used. **C)** Amplification curves of $5x10^8$ copies genomic HDV RNA mixed with $5x10^8$ (1:1), $5x10^7$ (1:10) and $5x10^6$ (1:100) copies of antigenomic HDV RNA and reverse transcribed with the antigenomic primer. The ct value for $5x10^6$ copies antigenomic HDV RNA is 31 and in accordance with the unspecific ct value for $5x10^8$ copies genomic HDV RNA also transcribed with the antigenomic primer, indicating that

antigenomic HDV RNA in a mixture with genomic HDV RNA can only be detected until a dilution of 1:100 (genomic: antigenomic). **D)** Serum control from a chronically HBV/HDV-infected USB mouse (2.4x10^5 copies/ml). Genomic HDV RNA was determined at a ct value of 31, whereas antigenomic HDV RNA and water were undetectable.

Suppl. figure 2

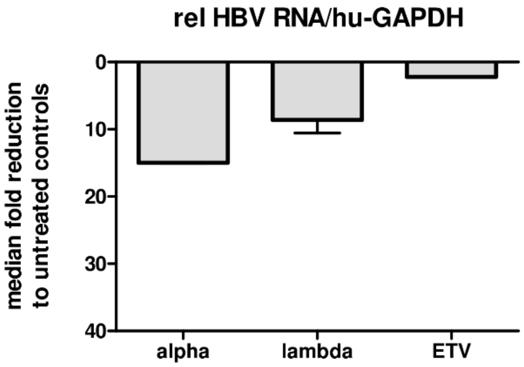

Suppl. figure 2. Changes of intrahepatic HBV activity upon treatment. Median fold reduction of total HBV RNA levels relative to hGAPDH in peg-IFNα, peg-IFNλ and ETV treated mice compared to untreated controls.

Suppl. figure 3

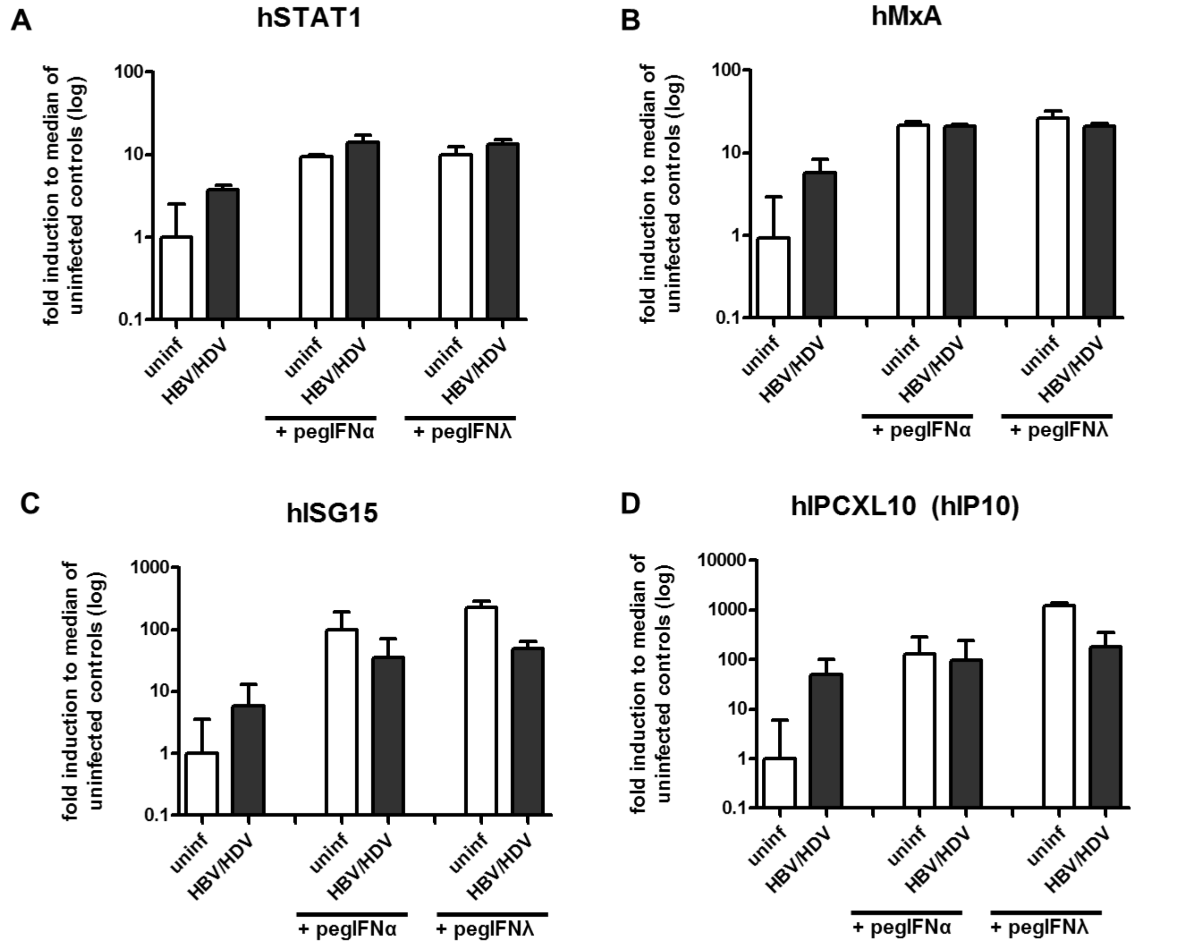

Suppl. figure 3. Interferon-stimulated genes. Expression of human specific signalling genes (hSTAT 1, **A**), interferon-stimulated genes (hMxA, **B**; hISG15, **C**) and cytokines (hCXCL10, **D**) in uninfected (white bars) and HBV/HDV co-infected mice (black bars), which were either untreated or treated once (uninfected) / 4 weeks (HBV/HDV-infected) with peg-IFNα or peg-IFNλ. All animals were sacrificed 24 hours after their last interferon injection. Expression levels are relative the median of two housekeeping genes (hGAPDH, hRPL30). Depicted is the log fold induction (median and range) from median of untreated uninfected controls.
